# The effects of joint aspiration and intra-articular corticosteroid injection on flexion reflex excitability, quadriceps strength and pain in individuals with knee synovitis: a prospective observational study

**DOI:** 10.1186/s13075-015-0711-5

**Published:** 2015-07-28

**Authors:** David Andrew Rice, Peter John McNair, Gwyn Nancy Lewis, Nicola Dalbeth

**Affiliations:** Health and Rehabilitation Research Institute, Auckland University of Technology, 55 Wellesley Street East, Auckland, 1010 New Zealand; Waitemata Pain Services, Department of Anaesthesiology and Perioperative Medicine, Waitemata District Health Board, 15 Shea Terrace, Auckland, 0740 New Zealand; Department of Medicine, University of Auckland, 2 Park Road, Auckland, 1023 New Zealand

## Abstract

**Introduction:**

Substantial weakness of the quadriceps muscles is typically observed in patients with arthritis. This is partly due to ongoing neural inhibition that prevents the quadriceps from being fully activated. Evidence from animal studies suggests enhanced flexion reflex excitability may contribute to this weakness. This prospective observational study examined the effects of joint aspiration and intra-articular corticosteroid injection on flexion reflex excitability, quadriceps muscle strength and knee pain in individuals with knee synovitis.

**Methods:**

Sixteen patients with chronic arthritis and clinically active synovitis of the knee participated in this study. Knee pain flexion reflex threshold, and quadriceps peak torque were measured at baseline, immediately after knee joint aspiration alone and 5 ± 2 and 15 ± 2 days after knee joint aspiration and the injection of 40 mg of methylprednisolone acetate.

**Results:**

Compared to baseline, knee pain was significantly reduced 5 (*p* = 0.001) and 15 days (*p* = 0.009) post intervention. Flexion reflex threshold increased immediately after joint aspiration (*p* = 0.009) and 5 (*p* = 0.01) and 15 days (*p* = 0.002) post intervention. Quadriceps peak torque increased immediately after joint aspiration (*p* = 0.004) and 5 (*p* = 0.001) and 15 days (*p* <0.001) post intervention.

**Conclusions:**

The findings from this study suggest that altered sensory output from an inflamed joint may increase flexion reflex excitability in humans, as has previously been shown in animals. Joint aspiration and corticosteroid injection may be a clinically useful intervention to reverse quadriceps muscle weakness in individuals with knee synovitis.

## Introduction

Quadriceps strength deficits of 20–40 % are typically observed in individuals with knee joint arthritis. This is partly due to muscle atrophy and partly due to ongoing neural inhibition that prevents the quadriceps from being fully activated by the central nervous system (i.e. a central activation deficit of the muscle) [1], a process known as arthrogenic muscle inhibition (AMI). Quadriceps AMI has been linked to joint effusion, inflammation and pain, joint laxity and articular structural damage [2]. The relative importance of these factors is unclear but it is generally accepted that AMI is caused by a change in the discharge of sensory receptors from the damaged knee joint [2, 3]. Abnormal joint afferent discharge may have powerful effects on the excitability of several central nervous system pathways that combine to prevent full activation of the quadriceps muscles (for a review see [4]). As well as being a direct cause of quadriceps weakness [1], AMI may contribute to muscle atrophy [2] and, in more severe cases, can prevent effective quadriceps strengthening [5], leading to persistent muscle weakness that is difficult to reverse. Ongoing quadriceps weakness is clinically important as it may impair dynamic knee joint stability [6], physical function [7, 8] and quality of life [8] as well as increasing the risk of further joint damage [9].

A number of experimental studies [10–12] have demonstrated that joint effusion is an important cause of AMI, with immediate reductions in quadriceps peak torque, electromyography (EMG) amplitude and H-reflex amplitude observed following the infusion of normal saline into healthy, uninjured knee joints. Similar experiments in animals [13, 14] have shown that joint infusion raises intra-articular pressure, stimulating stretch and pressure-sensitive articular mechanoreceptors and greatly enhancing the discharge of group II joint afferents.

While the aspiration of fluid from arthritic knee joints increases quadriceps peak torque, the subsequent infusion of local anaesthetic [15] or corticosteroid [16] has been shown to further enhance quadriceps muscle activation in these individuals. Moreover, recent studies have demonstrated that experimental knee pain leads to an immediate reduction in quadriceps peak torque [17, 18], voluntary muscle activation [17] and H-reflex amplitude [17], suggesting that joint nociception also plays an important role in quadriceps AMI. Both these mechanisms may be relevant to arthritic joint disease, where joint effusion is often perennial and the presence of inflammatory mediators substantially increases nociceptive discharge by ongoing peripheral sensitisation of group III and IV joint afferents (for a review see [19]).

The specific central nervous system pathways (e.g. spinal reflex and/or cortical pathways) by which abnormal joint afferent output leads to AMI are only partially understood. Several pathways may be affected by a change in joint afferent discharge and may thus be involved in AMI [2, 4]. One of the spinal reflex pathways thought to be involved is the flexion reflex [2]. The flexion reflex is a polysynaptic pathway that typically produces a pattern of flexor muscle facilitation and inhibition of extensor muscles such as the quadriceps [20]. Studies in animals have shown that flexion reflex is mediated, at least in part, by wide dynamic range neurons in the spinal cord [21], and that these interneurons receive excitatory sensory input from both mechanosensitive (group II) and nociceptive (group III and IV) knee joint afferents [22]. Furthermore, animal studies have demonstrated that experimentally induced knee joint arthritis greatly increases the excitability of the flexion reflex pathway [23, 24] and that this can be reversed by the subsequent injection of local anaesthetic into the joint [24]. Similarly, some cross-sectional studies in humans have shown that flexion reflex excitability is enhanced in patients with knee joint injury or pathology compared to healthy controls [25–27]. However, it has yet to be demonstrated that modifying joint afferent discharge alters flexion reflex excitability in humans and it is unknown whether this is accompanied by an improvement in quadriceps muscle strength.

Thus, the purpose of this prospective observational study was to explore the effects of knee joint aspiration and corticosteroid injection on flexion reflex threshold, quadriceps muscle strength and knee pain in individuals with arthritis and clinically active knee joint synovitis.

## Methods

### Participants

Sixteen participants with chronic knee joint arthritis (ten rheumatoid arthritis (RA), five osteoarthritis (OA), one psoriatic arthritis) who were attending outpatient rheumatology or physiotherapy clinics volunteered to take part in this study (Table 1). Participants were included if they had a clinically detectable knee joint effusion (brush/stroke test [28]) as well as other signs and symptoms of knee joint synovitis (pain on motion, heat and tenderness on palpation). They were excluded if they had received an intra-articular injection in the preceding 4 months, a cardiovascular condition precluding the performance of maximum effort strength tests, a documented loss of normal sensory function or a history of low back pain with associated neurological symptoms and signs. Participants were asked to refrain from caffeine, alcohol, nicotine, and strenuous exercise for 4 hours prior to each testing session [29]. In addition, they were asked not to take any analgesic medication in the 12 hours prior to each testing session but were otherwise told to continue taking their usual medication. Finally, participants were asked not to start any new medication or undertake any unaccustomed exercise during the testing period. Ethical approval for this study was given by the Northern Regional X Ethics Committee of New Zealand in accordance with the principles of the Declaration of Helsinki. All participants provided written informed consent.

**Table 1:** Participant characteristics

Age in years, mean (SD)	61.0 (13.7)
Height in metres, mean (SD)	1.65 (0.09)
Mass in kilograms, mean (SD)	81.9 (22.6)
Female, number (%)	11 (68.8)
Duration of pathology in months, mean (SD)	98.8 (78.3)
Type of pathology, number (%)	
Rheumatoid arthritis	10 (62.50)
Osteoarthritis	5 (31.25)
Psoriatic arthritis	1 (6.25)
Number of painful joints, mean (SD)	3 (3.25)

*SD* standard deviation

### Experimental protocol

Participants attended three testing sessions: baseline, 5 ± 2 days post intervention and 15 ± 2 days post intervention. For each participant, testing was conducted at the same time of day in each session to minimise circadian variations in flexion reflex threshold [30] and muscle strength [31]. At the baseline session, measures of knee pain, flexion reflex threshold and quadriceps peak torque were performed. A second baseline measure of flexion reflex threshold was undertaken 15 minutes later to ensure the stability of this measure. Thereafter, participants had their knee joint aspirated. Immediately following joint aspiration, flexion reflex threshold and then quadriceps peak torque were measured again. At the end of the baseline session, participants received an intra-articular injection of 40 mg methylprednisolone acetate. Participants were advised to rest the injected knee as much as possible in the first 72 hours after corticosteroid injection [32]. At 5 ± 2 days and 15 ± 2 days post intervention, knee pain, flexion reflex threshold and quadriceps peak torque were retested.

### Joint aspiration and corticosteroid injection

All participants had their knee joint aspirated while lying in a supine position with the knee resting in or near full extension. A 21-gauge needle was inserted into the superolateral aspect of the joint and as much synovial fluid as possible withdrawn from the knee. Where aspiration obtained notably less synovial fluid than expected from clinical examination (n = 5), a second aspiration was attempted using a medial approach. Following the post-aspiration measures of the dependent variables, 40 mg of methylprednisolone acetate (Depomedrol®) was injected into the knee joint. All joint injections were performed without local anaesthesia, under sterile conditions.

### Knee pain

Knee pain ratings were obtained using the P4. The P4 is a four-item instrument that gathers numerical pain ratings of pain in the morning, pain in the afternoon, pain in the evening and pain during activity [33] over the last 2 days. The P4 has been validated in individuals with arthritis [34] and has superior reliability and responsiveness compared to single-item numerical pain rating scales [33].

### Flexion reflex threshold

A Nicolet bar electrode (stimulating electrode) with 9 mm gold cups and 30 mm interelectrode distance (Weaver and Company, Aurora, CO, USA) was secured under the medial arch of the foot and covered with an elastic bandage. The stimulating electrode was placed in a standardised position 2 cm proximal to the first metatarsophalangeal joint and directly underneath the first metatarsal on the plantar aspect of the foot, with the anode positioned distally. For recording purposes, bipolar Ag-AgCl disc electrodes with an interelectrode distance of 2.2 cm were placed over the biceps femoris muscle of the ipsilateral leg, with the inferior edge positioned 10 cm above the popliteal crease [29]. A ground electrode was placed on the anterior surface of the ipsilateral tibia. Prior to placement of both the stimulating and recording electrodes, the electrode sites were shaved, abraded and cleansed with an alcohol wipe to reduce skin impedance. EMG signals were amplified (1000×), filtered (10–1000 Hz; AMT-8 amplifier, Bortec Biomedical, Calgary, AL, Canada), and sampled at 2000 Hz (Micro 1401, Cambridge Electronic Design, Cambridge, UK).

Once the stimulating and recording electrodes were in place, participants stood on a raised, carpeted wooden block (150 × 40 × 26 cm) and were asked to support their weight through their contralateral leg and arms while holding onto the back of a chair for balance. The stimulated leg hung in a relaxed position without touching the ground. Participants were asked to look straight ahead during testing and to keep the muscles in their stimulated leg completely relaxed. Muscle relaxation was ensured by continuous monitoring of biceps femoris surface EMG in real time on an oscilloscope (Textronix TDS2014B, Beaverton, OR, USA). The reliability of testing the flexion reflex threshold in this manner has been established in our laboratory [35]. Electrocutaneous stimuli were then delivered to the medial arch of the foot using a DS7A constant current stimulator (Digitimer Ltd, Welwyn Garden City, UK) controlled by a specialised software programme (Signal 3, Cambridge Electronic Design, Cambridge, UK). Each stimulus train consisted of five rectangular pulses of 1 ms pulse width with a 3 ms interpulse interval (17 ms total duration) [36]. Stimuli were separated by a random interstimulus interval of 8–12 seconds [29]. Prior to threshold testing participants were familiarised with the stimulation procedures by receiving a series of 15 stimuli of varying intensity. The flexion reflex threshold was then determined using an up-down staircase method [29]. Specifically, the stimulation intensity was increased from 0 mA in 4 mA increments until a flexion reflex was observed. Once a flexion reflex response was observed, the intensity was decreased in 2 mA steps until a flexion reflex was no longer evident. The stimulation intensity was then increased and decreased four more times in 1 mA increments until a flexion reflex appeared twice and disappeared twice. These final four stimulation intensities were recorded and averaged to calculate flexion reflex threshold. The presence of a flexion reflex response was defined using a flexion reflex interval peak z-score of biceps femoris EMG activity, derived using the formula [29]:

$$ Z- score = \left( flexion\  reflex\ int erval\  peak\ \hbox{--}\ baseline\  mean\right)\ /\  baseline\ s tan dard\  deviation $$

The baseline mean and standard deviation of biceps femoris EMG activity were recorded from a 65 ms period (−70 to −5 ms) prior to stimulation. The flexion reflex interval peak refers to the peak EMG activity of the biceps femoris muscle during the post stimulation window of 85–150 ms. This window was chosen to avoid signal contamination via non-nociceptive reflexes and startle responses [37]. A z-score of 10.32 or greater was considered a true flexion reflex response [29].

### Quadriceps peak torque

Participants were seated in a custom-designed chair with the hips and knees flexed to 90°. Straps were firmly secured over the distal third of the thigh and across the chest to limit extraneous movement. A rigid strap was secured around the ankle, slightly superior to the malleoli. This was coupled in series to a uniaxial load cell (Precision Transducers, Auckland, New Zealand), aligned horizontally with the ankle joint. All participants were asked to perform isometric maximum voluntary contractions (MVCs) of their quadriceps by pushing as hard as possible against the ankle strap. Prior to maximum effort contractions, a series of four submaximal quadriceps contractions (25 %, 50 %, 50 % and 75 % of perceived maximum effort) were performed, with a 1-minute rest given between each contraction. These were performed as “warm-up” contractions in an attempt to minimise the risk of participant injury and facilitate muscle force production during subsequent maximum effort contraction [38, 39]. Thereafter, a 2-minute rest was given before a set of three (6-second) MVCs were performed. Participants received visual feedback of their force trace and a consistent level of strong verbal encouragement during each contraction. A 2-minute rest period was given between each maximum effort contraction. In the event that the peak force (N) produced during the MVCs continued to increase with each subsequent trial (six participants), a fourth, and in some cases a fifth, contraction was performed until force plateaued or decreased. This was done in an effort to elicit a true maximum effort from each individual. Force signals were recorded from the load cell during each contraction, where they were amplified (×100), sampled (1000 Hz) and stored on a computer for later analysis. At each measurement interval, peak isometric quadriceps strength was calculated as the highest force (N) produced during any of the three to five MVCs. The length of the lever arm was measured from the lateral epicondyle of the femur to the centre of the ankle strap, which was parallel to the load cell. Lever arm length (m) was then multiplied by peak isometric force (N) to calculate peak torque (Nm).

### Statistical analysis

To assess whether the dependent variables conformed to a normal distribution Shapiro-Wilk tests were completed. One-way repeated measures ANOVAs were undertaken to analyse differences in flexion reflex threshold and quadriceps peak torque over time (baseline, day 5, and day 15). Where the assumption of sphericity was violated, Greenhouse-Geisser corrections were utilised. As our a priori hypotheses were that flexion reflex threshold and quadriceps peak torque would increase following aspiration and corticosteroid injection, planned contrasts were used to assess the differences between the first (baseline 1) and each subsequent measure. As the P4 pain scores were not normally distributed, a Friedman’s test was used to examine changes in knee pain over time. Thereafter, Wilcoxon signed-rank tests were utilised to compare baseline knee pain to knee pain on day 5 and day 15. The alpha level for all statistical procedures was set to 0.05.

## Results

Baseline characteristics of the participants are provided in Table 1. The mean volume of fluid aspirated from the knee joint was 24 ± 27 ml. Quadriceps peak torque was collected from all 16 participants. Complete data sets for measures of flexion reflex threshold could only be obtained from 11 of the 16 participants. One participant with RA was unable to support their body weight on the contralateral leg due to painful synovitis in the first metatarsophalangeal joint. Equipment failure prevented flexion reflex threshold being collected at day 5 for two participants and day 15 for two participants. All participants acted appropriately in respect to the instructions regarding exercise changes and medication use during the testing period.

### Knee pain

**Fig. 1:**
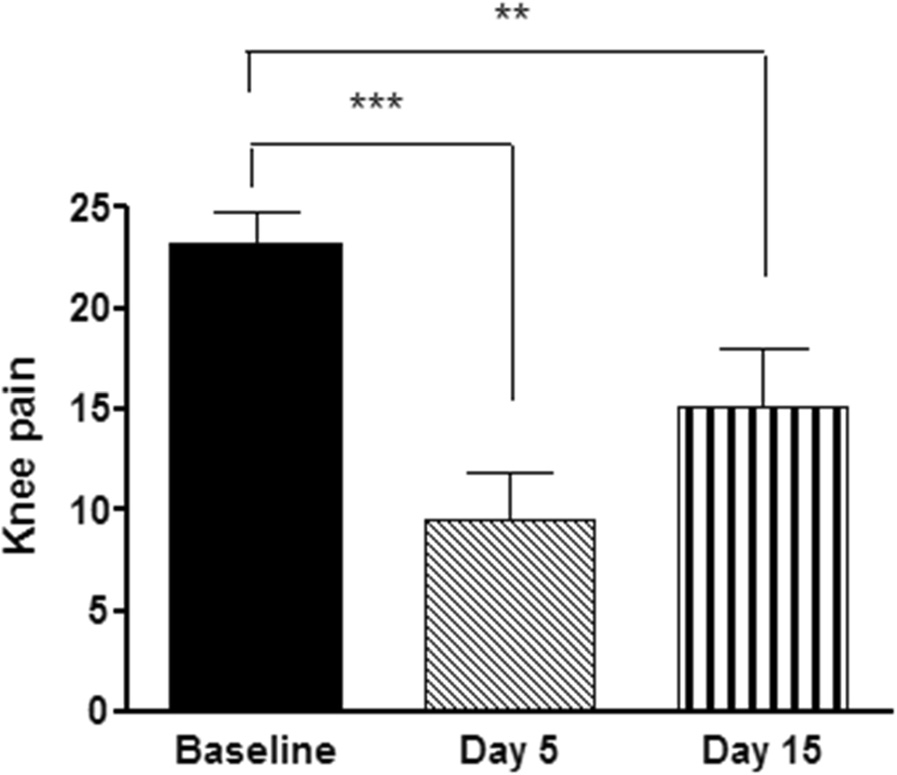
Knee pain as measured on the P4 instrument before and after knee joint aspiration and corticosteroid injection. Baseline = before intervention. Day 5 = 5 ± 2 days after knee joint aspiration and corticosteroid injection. Day 15 = 15 ± 2 days after knee joint aspiration and corticosteroid injection. **= significant change from baseline (*p* <0.01). ***= significant change from baseline (*p* = 0.001). Data are means and one standard error of the mean

There was a significant change in knee pain over time (*p* <0.001) (Fig. 1). Compared to baseline, there was a significant reduction in knee pain at 5 ± 2 days (*p* = 0.001) and 15 ± 2 days (*p* = 0.009) after aspiration and corticosteroid injection.

### Flexion reflex threshold

**Fig. 2:**
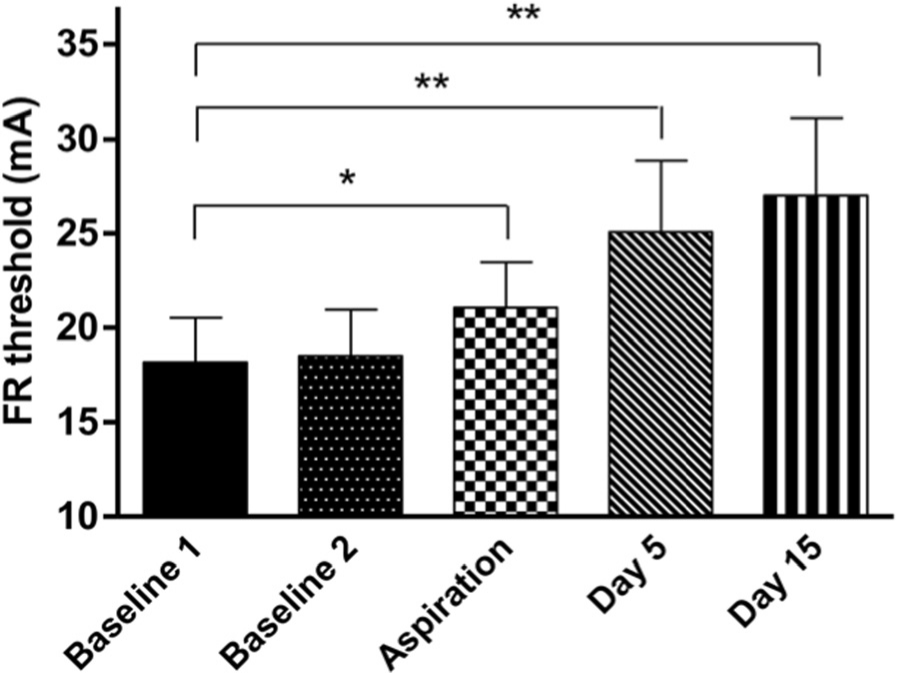
Flexion reflex (FR) threshold before (baseline 1 and baseline 2) and after knee joint aspiration and corticosteroid injection. mA = milliamps. Aspiration = following joint aspiration only. Day 5 = 5 ± 2 days after knee joint aspiration and corticosteroid injection. Day 15 = 15 ± 2 days after knee joint aspiration and corticosteroid injection. *= significant change from baseline 1 (*p* <0.05). **= significant change from baseline 1 (*p* <0.01). Data are means and one standard error of the mean

A significant change in flexion reflex threshold was observed over time (*p* = 0.002) (Fig. 2). Flexion reflex threshold was significantly higher than baseline after aspiration alone (*p* = 0.009) and at 5 ± 2 days (*p* = 0.01) and 15 ± 2 days (*p* = 0.002) after aspiration and corticosteroid injection. No significant difference was found between the baseline 1 and baseline 2 measures of flexion reflex threshold (*p* = 0.479).

### Quadriceps peak torque

**Fig. 3:**
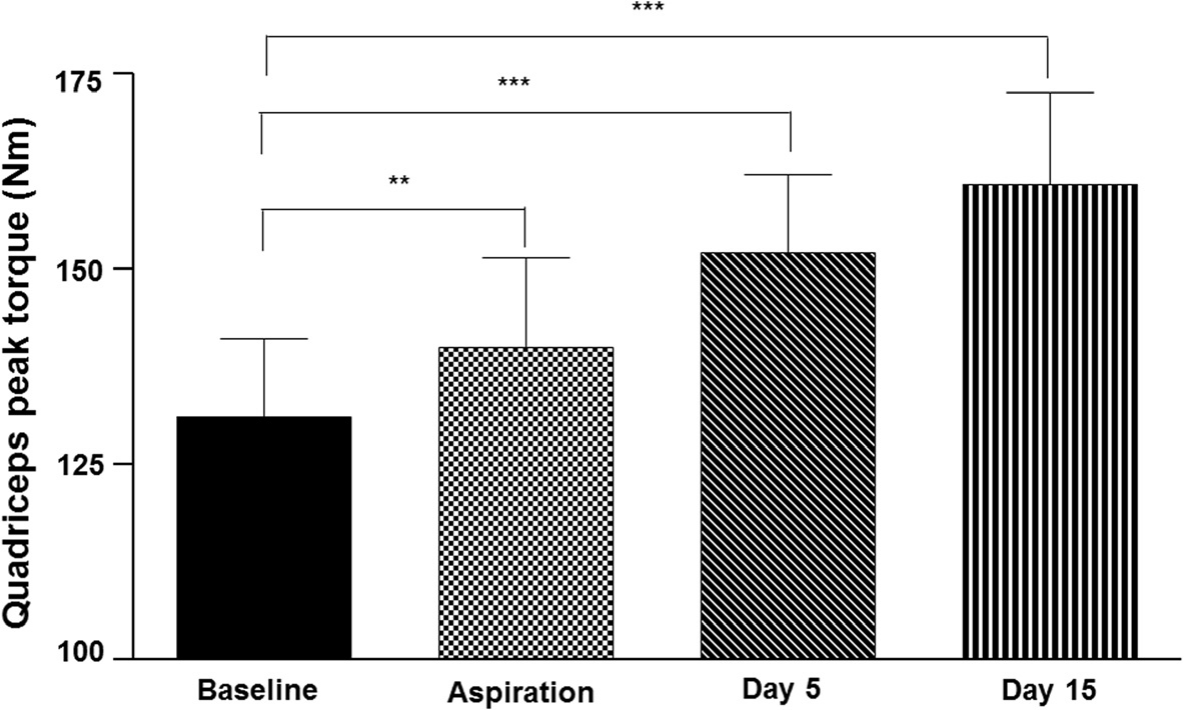
Quadriceps peak torque at baseline and following knee joint aspiration and corticosteroid injection. Nm = Newton metres. Aspiration = following joint aspiration only. Day 5 = 5 ± 2 days after knee joint aspiration and corticosteroid injection. Day 15 = 15 ± 2 days after knee joint aspiration and corticosteroid injection. **= significant change from baseline (*p* <0.01). ***= significant change from baseline (*p* ≤0.001). Data are means and one standard error of the mean

There was a significant change in quadriceps peak torque over time (*p* <0.001) (Fig. 3). Quadriceps peak torque was significantly higher than baseline after aspiration alone (*p* = 0.004) and at 5 ± 2 days (*p* = 0.001) and 15 ± 2 days (*p* <0.001) after aspiration and corticosteroid injection.

## Discussion

The findings of this study provide evidence that altered knee joint afferent discharge modifies flexion reflex excitability in humans, as has previously been demonstrated in animals [24]. We observed an increase in flexion reflex threshold immediately after the aspiration of fluid from the joint and a larger increase 5 and 15 days after subsequent corticosteroid injection. Knee joint effusion is an established cause of AMI, with experimental joint infusion leading to immediate decreases in quadriceps strength and muscle activation that can be reversed or prevented by the intra-articular injection of local anaesthethic. Recent findings [40] have failed to demonstrate a role of cortical pathways in this inhibition. However, experimental joint infusion markedly suppresses the quadriceps H-reflex amplitude [11, 41] (a measure of spinal reflex excitability) and has been shown to enhance group I non-reciprocal (Ib) inhibition of the quadriceps motoneuron pool, both at rest and during voluntary muscle contraction [42]. In the current study, the reduction in flexion reflex excitability following aspiration alone is a novel finding, suggesting that joint effusion may enhance flexion reflex excitability as well as increasing the excitability of other spinal reflex inhibitory pathways such as group I non-reciprocal (Ib) inhibition [4].

In humans, experimental joint effusion rarely evokes sensations of pain [12], suggesting that that effusion alone is unlikely to stimulate a significant number of nociceptive (group III and IV) afferents. Furthermore, in animal studies, the infusion of saline into undamaged knee joints failed to increase flexion reflex excitability [23]. However, the afferent response to effusion may differ markedly in arthritic joints, where the presence of inflammatory mediators directly activates a portion of group III and IV joint afferents while at the same time sensitising these afferents to previously innocuous mechanical stimuli such as an increase in intra-articular pressure [43]. Furthermore, central sensitisation of wide dynamic range interneurons may increase the synaptic efficacy of group II joint afferents acting on the flexion reflex pathway, as these interneurons are thought to be involved in mediating the flexion reflex response [21, 44]. Thus, in the presence of synovitis, it is likely that aspiration reduces the discharge of group II, III and IV knee joint afferents, all of which have the potential to influence flexion reflex excitability.

The increase in flexion reflex threshold 5 and 15 days after aspiration and corticosteroid injection may be explained by the anti-inflammatory effects of corticosteroid injection and subsequent reduction in the discharge of group III and IV afferents. Locally administered corticosteroid has been shown to have a direct inhibitory effect on the ability of group IV afferents to generate action potentials [45]. Furthermore, corticosteroid injections are known to strongly suppress local joint inflammation [46] and thus can be expected to both reduce the activation of chemosensitive free nerve endings and raise the activation threshold of group III and IV joint afferents affected by peripheral sensitisation. The subsequent reduction in articular sensory output may reduce spatial facilitation between nociceptive cutaneous afferents in the foot and joint afferents [20], increasing the flexion reflex threshold. Alternatively, it is possible a reduction in nociceptive input from the arthritic joint partially reverses neuroplastic changes in the spinal cord interneurons, mediating the flexion reflex response. Such a reduction in central nervous system excitability following the removal or blockade of peripheral afferent input has been observed previously [47–49]. Finally, it is possible that aspiration and corticosteroid injection led to a change in descending inhibition and/or facilitation of the flexion reflex from supraspinal structures. In this regard, Herrero et al. [50] have shown that supraspinal pathways are at least partly responsible for the increase in flexion reflex excitability observed in cats during experimental knee arthritis. Furthermore, in humans, a reversal in the dysfunction of conditioned pain modulation pathways has been shown after both knee [48] and hip [51] arthroplasty. As conditioned pain modulation is at least partly determined by supraspinal inhibition of wide dynamic range neurons [52], it is possible that reducing the nociceptive barrage from the damaged joint may enhance descending inhibition and/or reduce descending facilitation of the flexion reflex pathway [48, 51].

An important finding from the current study is that aspiration and corticosteroid injection led to a notable increase in quadriceps peak torque in individuals with chronic knee joint arthritis. These findings support the earlier work of Geborek et al. [16], who reported a 9 Nm increase in quadriceps peak torque immediately after knee joint aspiration and a larger 26 Nm increase 14 days after subsequent corticosteroid injection in RA patients. Similarly, we observed a 9 ± 10 Nm increase in torque immediately following aspiration and a 31 ± 19 Nm increase by day 15, an average 25 % increase in quadriceps peak torque from baseline levels. Such a rapid change in muscle strength is likely to reflect enhanced neural activation of the muscle as changes in muscle structure such as fibre hypertrophy take longer than 15 days to occur, even with heavy resistance training [53]. This is supported by our finding of an immediate increase in quadriceps peak torque following knee joint aspiration, which indicates the presence of quadriceps AMI in our participants.

Quadriceps AMI is common in patients with RA [54] and OA [55]. Interestingly, the mean 25 % increase in quadriceps peak torque we observed 15 days after aspiration and corticosteroid injection is of a similar magnitude to the reported change in quadriceps strength produced by 5–6 months of high-intensity resistance training in some studies [56, 57]. While AMI does not always preclude quadriceps strength gains [54], even mild AMI can restrict their magnitude as a portion of the muscle cannot be activated [58, 59]. Recent work in patients with OA suggests that combining transcutaneous electrical nerve stimulation (an intervention known to reduce AMI [60]) with resistance training produces greater improvements in quadriceps activation and strength than resistance training alone [59]. Furthermore, in patients with OA, the combination of non-steroidal anti-inflammatory drugs (NSAIDs) and resistance training has been shown to lead to greater increases in quadriceps strength compared to resistance training and placebo tablets [58]. Given the approximately 25 % increase in quadriceps strength we observed in the current study, it may be that aspiration and corticosteroid injection followed by a period of resistance training will lead to greater long-term quadriceps strength gains than resistance training alone in patients with knee joint synovitis. This should be investigated in future studies.

Limitations of this study include the relatively small sample size and diversity in the underlying joint pathology. However, all participants were included due to clinically detectable synovitis and, despite the sample size, significant changes were observed in all the dependent variables. While we consider it more likely that our findings are explained by a reduction in abnormal joint afferent output, the pre-post design of our study and lack of a control group means we cannot rule out non-specific treatment effects such as placebo contributing to the observed effects. However, corticosteroid injection is known to have potent anti-inflammatory effects at the injected joint; reducing joint effusion [46], mast cell [61] and macrophage [61] numbers; suppressing T cell production [62] and reducing both pro-inflammatory cytokine [62] and leukotriene [63] expression in the synovium. Furthermore, the between-session reliability of the quadriceps peak torque [64] and flexion reflex threshold [35] has been established, placebo injection has previously been shown to have no effect on quadriceps strength [65] and there is level Ia evidence that corticosteroid injection has a significant therapeutic effect over and above placebo injection [66].

## Conclusions

In this study, aspiration and corticosteroid injection of chronic arthritic knee joints led to a significant increase in flexion reflex threshold, suggesting that aberrant joint afferent output due to effusion and inflammation enhances flexion reflex excitability in humans as has previously been observed in animals. Furthermore, aspiration and corticosteroid injection led to a mean 25 % increase in quadriceps peak torque after 15 days, suggesting a clinically important reduction in quadriceps AMI. Future research in patients with knee synovitis should explore whether quadriceps resistance training can be enhanced by the prior aspiration and injection of corticosteroid into the joint.

## Abbreviations

AMI: arthrogenic muscle inhibition
EMG: electromyography
MVC: maximum voluntary contraction
NSAIDs: non-steroidal anti-inflammatory drugs
OA: osteoarthritis
RA: rheumatoid arthritis
